# Supplementary material for: Complexome profiling of the Chlamydomonas psb28 mutant reveals TEF5 as an early PSII assembly factor
Source: Plant Cell. 2025 Mar 17;37(6):koaf055. doi: 10.1093/plcell/koaf055 (PMC12164590; doi:10.1093/plcell/koaf055)
Supplement: koaf055_Supplementary_Data [file koaf055_supplementary_data.zip › psb28_interactive.zip!/plots/intensity/qualityPlots/peptides/_Cre07.g331700.t1.1.html]

### Cre07.g331700.t1.1

Trivial name:   
SeqCoverage [median]: 1.50  
Mass: 123.56 kDa  
  
 

| Sequence | MissedCleavage | Mass [Da] | wtIntensity | muIntensity | Score |
| --- | --- | --- | --- | --- | --- |
| DSSPDTSARYR | 1 | 1253.56 | 1088.65 | 1725.69 | 42.74 |
| VGQTWWTK | 0 | 1004.51 | 0.00 | 1656.25 | 68.92 |
|  |  |  | 1088.7 | 3381.9 |  |
